# A Novel Nomogram for Predicting Gestational Diabetes Mellitus During Early Pregnancy

**DOI:** 10.3389/fendo.2021.779210

**Published:** 2021-12-09

**Authors:** Mei Kang, Hui Zhang, Jia Zhang, Kaifeng Huang, Jinyan Zhao, Jie Hu, Cong Lu, Jiashen Shao, Jianrong Weng, Yuemin Yang, Yan Zhuang, Xianming Xu

**Affiliations:** ^1^ Clinical Research Center, Shanghai General Hospital, Shanghai Jiaotong University School of Medicine, Shanghai, China; ^2^ Department of Obstetrics and Gynecology, Shanghai General Hospital, Shanghai Jiaotong University School of Medicine, Shanghai, China; ^3^ Department of Obstetrics and Gynecology, Suining County People’s Hospital, Xuzhou, China; ^4^ Department of Clinical Laboratory, Shanghai General Hospital, Shanghai Jiaotong University School of Medicine, Shanghai, China; ^5^ Nuclear Medicine Department, Shanghai General Hospital, Shanghai Jiaotong University School of Medicine, Shanghai, China

**Keywords:** gestational diabetes mellitus, B lymphocytes, IgA, risk factors, nomogram

## Abstract

**Objective:**

Gestational diabetes mellitus (GDM) is a serious threat to maternal and child health. However, there isn’t a standard predictive model for the disorder in early pregnancy. This study is to investigate the association of blood indexes with GDM and establishes a practical predictive model in early pregnancy for GDM.

**Methods:**

This is a prospective cohort study enrolling 413 pregnant women in the department of Obstetrics and Gynecology in Shanghai General Hospital from July 2020 to April 2021.A total of 116pregnantwomen were diagnosed with GDM during the follow-up. Blood samples were collected at early trimester (gestational weeks 12-16) and second trimester(gestational weeks 24-26 weeks). A predictive nomogram was established based on results of the multivariate logistic model and 5-fold cross validation. We evaluate the nomogram by the area under the receiver operating characteristic curve (AUC), calibration curves and decision curve analysis (DCAs).

**Results:**

Significant differences were observed between the GDM and normal controls among age, pre-pregnancy BMI, whether the pregnant women with complications, the percentage of B lymphocytes, fasting plasma glucose (FPG), HbA1c, triglyceride and the level of progesterone in early trimester. Risk factors used in nomogram included age, pre-pregnancy BMI, FPG, HbA1c, the level of IgA, the level of triglyceride, the percentage of B lymphocytes, the level of progesterone and TPOAb in early pregnancy. The AUC value was 0.772, 95%CI (0.602,0.942). The calibration curves for the probability of GDM demonstrated acceptable agreement between the predicted outcomes by the nomogram and the observed values. DCA curves showed good positive net benefits in the predictive model.

**Conclusions:**

A novel predictive nomogram was developed for GDM in our study, which could do help to patient counseling and management during early pregnancy in clinical practice.

## Introduction

Gestational diabetes mellitus (GDM) is the most common metabolic syndrome defined as diabetes diagnosed in the second and third trimester of pregnancy, which characterized as hyperglycemia (1). The prevalence of GDM in increasing trend at present varies from 1% to 30%all over the world (2, 3). GDM brings great risks to both the mother and developing fetus (4). For the mother, it increases the risk of cesarean section, preeclampsia and the type 2 diabetes after delivery. For the fetus, it makes them face the risk of adverse pregnancy outcomes, such as malformation, preterm labor, and macrosomia even dystocia etc. (5, 6). In the long term, the increase hyperglycemia is associated with the rising incidence of obesity and metabolic syndrome in adolescence (7).

The etiology of GDM is very complicated. At present, it is generally accepted that insulin resistance and the dysfunction of β cells contribute a lot to GDM. In normal pregnancy, the level of insulin resistance enhances with the increase of gestational age. Then the islets secret more insulin to compensate for the growing insulin resistance. However, in pregnant women with GDM, because of the greatly increased insulin resistance, the production of insulin is insufficient. Therefore, the blood glucose goes up. Owing to the similar pathology with type 2 diabetes mellitus, GDM is regarded as the early stage of T2DM (8).

Recently, the view that GDM, characterized by insulin resistance, is a chronic low-grade inflammation has been widely recognized. Chronic metaflammation caused by metabolic nutrients and inflammatory factors decrease the insulin sensitivity and destroy β cells (9). Many immune cells and factors are involved in this inflammation, including macrophages, T lymphocytes, B lymphocytes, TNF-α, IFN-γ etc. (10). Since Daniel A Winer et al. reported that B lymphocytes promoted insulin resistance through modulation of T cells and IgG in 2011, the role of B lymphocytes in the initiation of insulin resistance process in diabetes mellitus has been gradually concerned (11). Our previous case-control study demonstrated that the percentage of B lymphocytes and the IgA antibodies produced by them in pregnant women with GDM (n=124) are both higher than non-diabetic pregnant women (n=168), in addition, the percentage of B lymphocytes in pregnant women with GDM is positively correlated with insulin resistance (12). Hence, we suspected the percentage of B lymphocytes and IgA could become potential predictors of GDM.

Besides, multiple factors have been reported in previous publications including age, BMI before pregnancy, life style, environmental and psychosocial factors, disorder of lipid metabolism (13–15), the placental hormones (16), the function of thyroid (17, 18). A clinical study involving 2211 pregnant women showed that TPOAb was an independent risk factor for GDM with assisted pregnancy (19). Another clinical research in 2021 revealed that increased FT3, TPOAb, TGAb in the first and second trimesters significantly increased GDM risk. Thyroid antibodies were even associated with postpartum glucose metabolism in GDM patients (20). Unfortunately, until now clinicians lack a standard model to predict GDM in early pregnancy. Therefore, we designed a prospective study to identify the independent risk factors for GDM presented as a novel nomogram for clinical use.

## Materials and Methods

### Study Population

We have screened 672 pregnant women who established pregnancy files in the department of Obstetrics and Gynecology in Shanghai General Hospital from July 2020 to April 2021. Only singleton pregnancies were included. Sociodemographic characteristics, pre-pregnancy BMI, parity and the blood indexes of the percentage of B lymphocytes, the level of IgA, fasting plasma glucose, fasting blood insulin, HbA1C, triglyceride, cholesterol, estrogen, progesterone, free triiodothyronine (FT3), free thyroxine(FT4), thyroid stimulating hormone (TSH), thyroid peroxidase antibody(TPOAb) and thyroglobulin antibody (TGAb) both in early pregnancy (12-16 gestational weeks) and the second trimester (24-26 gestational weeks when 75g OGTT test was performed) were recorded. Women were excluded if they were younger than 18 years old or older than 40 years old and if they had Type I diabetes mellitus or Type 2 diabetes mellitus diagnosed before pregnancy, autoimmune disorders, recent history of inflammation, cancer or psychiatric disorders. The PGDM pregnant women, whose (FPG) fasting plasma glucose ≥7.0mmol/l or 2h PBG (plasma glucose) ≥11.1mmol/l or RBG (random blood glucose) ≥11.1mmol/l or HbA1c (glycated hemoglobin) ≥6.5% during pregnancy were excluded. On the basis of diagnosis criteria from IADPSG in 2015, the cutoff of values was 5.1mmol/l for fasting plasma glucose, 10.0mmol/l for 1 hour plasma glucose, 8.5mmol/l for 2 hour plasma glucose. Informed consent forms were obtained from all the participants.

### Laboratory Tests

All the blood samples were collected by trained nurses in the department of Obstetrics and Gynecology, and the blood tests were performed by trained practitioners in the clinical laboratory in Shanghai General Hospital. The blood samples were withdrawn from patients after 8 hours of fasting.

We used high pressure liquid chromatography to detect glycosylated hemoglobin [fully automatic glycosylated hemoglobin analyzer G8 (Tosoh, Japan)]; GPO-PAP method to detect triglycerides; enzyme analysis method to detect cholesterol, and the hexokinase method (Glucose determination kit) to detect blood glucose [fully Automatic biochemical analyzer ADVIA2400 (Siemens, Germany)]. Direct chemiluminescence method was used for insulin detection [Automatic immunoassay analyzer ADVIA Centaur XP (Siemens, Germany)]. Solid-phase coating one-step sandwich method and radioimmunoassay were used for detection of free triiodothyroxine Amino acid [(FT3) Tianjin Xiehe T3247. Human thyroid-stimulating hormone [(TSH) Tianjin Xiehe T4247], two-step solid-phase coating method and radioimmunoassay were used to detect free thyroxine [(FT4) Tianjin Xiehe T60247]. We used secondary antibody polyethylene glycol precipitation method and radioimmunoassay to detect progesterone [Tianjin Xiehe T31247], and estradiol [Tianjin Xiehe T33247]. Electrochemiluminescence and competition method to detect thyroglobulin antibody [(TGAb) Roche Diagnosis 54235600] and anti-thyroid peroxidase antibody [(TPOAb) Roche diagnosis 53886300] were used.

### Lymphocytes Subsets

A 50 µL aliquot of whole blood was collected in the tube: one for T-cell B-cell and NK cells, followed by the addition of 20 µL four-color reagent CD3-FITC/CD16+56-PE/CD45-PerCP/CD19-APC (2021010100 for IVD, Mindray) to T-,B- and NK-cells. The antibodies and the whole blood were incubated for 15 mins in the dark, with gentle agitation at room temperature, followed by addition of 450 µL 1× Hemolysins (M-30PCFL, Mindray) to the tube and incubated for 5 mins at room temperature with the soft vortex. Finally, the tube was placed on a Bricyte E6 (Mindray) after calibration by SpheroTM Supra Rainbow Midrange Fluorescent Particles (Spherotec, No. SRCP-35- 2A, USA). Figures and analyses were performed using the FACSDIVA 6.1 software (BD Bioscience, San Joes, CA, USA).

### Immunoglobulin Detection

Immunoglobulin IgA was detected by the Immunoglobulin Quantification Kit (Beckman Coulter USA) according to the manufacturer’s instructions using AU5800 equipment (BECKMAN COULTER).

### Sample Size Calculation

With the GDM prevalence of 15% and Nagelkerke R^2^ of 0.5, targeting a shrinkage of 10% and 10 candidate predictor parameters, a mean absolute prediction error (MAPE) of 0.05, a sample size of at least 332 participants (about 50 events) is required to develop the GDM prediction model in the development dataset (21).

### Statistical Methods

Continuous variables were expressed as mean and standard deviation. Student *t* test was performed for normally distributed variables, or Mann Whitney U test otherwise. Categorical variables were presented as frequencies and percentages, and analyzed by Chi-square test or Fisher’s exact test. Multivariable analysis was performed using logistic regression model with 5-fold cross-validation. The final model trained on the training cohort was validated in the validation dataset. The regression coefficient were applied to the corresponding factors. Confirmed variables included in the final model met the following criterion. First, variables selected by the multivariable analysis [(backward elimination (Wald)]; second, clinically considered relevant variables were included. A predictive nomogram was built based on the outcome of multivariate logistic analysis, which included age, pre-pregnancy BMI, FPG, HbA1c, the level of IgA, the level of triglyceride, the percentage of B lymphocytes, the level of progesterone and TPOAb in early pregnancy. We evaluate the nomogram by the area under the receiver operating characteristic curve (AUC), calibration curves (Hosmer-Lemeshow test was used to assess the goodness of fit) and decision curve analysis (DCAs). *P* value<0.05 (two-tailed) was considered statistically significant. Data analyses were performed using R 4.0.5 software (the R Foundation for Statistical Computing, Vienna, Austria).

## Results

### Patient Characteristics and Univariable Analysis

We included 413 pregnant women in the cohort, then identified 116 GDM at the second trimester. The baseline characteristics are shown in **Table 1**, the average age of pregnant womenin GDM cases (31.1 ± 4.6 year-old) was significantly older than normal women (29.4 ± 3.6 year-old). GDM cases have significantly higher pre-pregnancy BMI and incidence of complications especially hypothyroidism and subclinical hypothyroidism than normal group (all *p*<0.05). The percentage of B lymphocytes in GDM patients was higher than normal ones (*p* =0.032), yet there was not statistical difference in the level of serum IgA between these two groups. The fasting plasma glucose, fasting insulin, HbA1c, and triglyceride in GDM cases were higher than those in normal women (all *p*<0.05). The level of estrogen and progesterone were both lower in GDM cases (all *p*<0.05).There was no noticeable difference in the level of thyroid hormones between two groups.

**Table 1:** Baseline patient characteristics and univariable analysis results*.

	GDM (n = 116)	Normal (n = 297)	*p* value
Age (year)	31.1 ± 4.6	29.4 ± 3.6	<0.001
Pre-pregnancy BMI (kg/m^2^)	21.79 ± 3.46	23.39 ± 4.02	<0.001
Parity [n(%)]	45/116 (38.6)	115/297 (38.7)	0.989
Complications [n(%)]	38/116 (32.8)	56/297 (18.9)	0.002
Hypothyroidism[n(%)]	27/116(23.3)	40/297 (13.5)	0.015
GDM history [n(%)]	2/58 (3.4)	0/110 (0)	0.118
CD19+B lymphocyte [n(%)]	11.43 (3.87)	10.56 (3.49)	0.032
IgA (g/L)	2.05 ± 0.63	2.15 ± 0.80	0.256
Fasting plasma glucose (mmol/l)	5.20 ± 0.48	4.95 ± 0.36	<0.001
Fasting insulin (mmol/L)	58.49 ± 29.06	45.86 ± 20.37	<0.001
HbA1c (%)	5.08 ± 0.35	4.91 ± 0.28	<0.001
Triglyceride (mmol/L)	1.83 ± 0.76	1.52 ± 0.60	<0.001
Cholesterol (mg/dL)	5.10 ± 0.91	5.11 ± 0.86	0.842
Estrogen (pg/mL) [Median (IQR)]^#^	458.07(322.44, 657.28)	475.37(328.84, 696.78)	0.016
Progesterone (ng/ml)	71.57 ± 20.99	77.80 ± 19.93	0.006
Free triiodothyronine (pmol/l)	4.91 ± 1.83	4.94 ± 4.00	0.944
Free thyroxine (pmol/l)	13.76 ± 5.33	14.44 ± 5.78	0.287
Thyroid stimulating hormone (mIU/ml)	1.79 ± 1.30	1.69 ± 3.50	0.776
Thyroid peroxidase antibody (TPOAb) [n(%)]	14/116 (12.1)	25/297 (8.4)	0.254
Thyroglobulin antibody (TGAb) [n(%)]	9/107 (7.8)	27/297 (9.1)	0.666

*Normally distributed continuous variables were described as mean ± Standard Deviation (SD); ^#^non-normal distribution parameters were presented using median and inter-quartile range (IQR).

### Multivariable Analysis and Model Construction

Both statistically significant factors in multivariable analysis and clinical considered relevant factors were used in the final model including age (OR 1.100, 95% CI 1.020-1.180), pre-pregnancy BMI (OR 1.080, 95% CI 1.002-1.170), parity (yes v.s. no: OR 0.410, 95% CI 0.220-1.330), FPG (OR 4.260, 95% CI 2.130- 8.490), HbA1c (OR 4.480, 95% CI 1.830- 11.010), the level of IgA (OR 0.740, 95% CI 0.500-1.100), the percentage of B lymphocytes (OR 1.080, 95% CI1.004-1.160), the level of triglyceride (OR 1.480, 95% CI 1.010-2.160), the level of progesterone (OR 0.990, 95% CI 0.980-1.001) in early pregnancy and TPOAb (positive vs. negative: OR 1.010, 95% CI 0.370-2.740) (**Table 2**).

**Table 2:** Multivariable logistic model of probability of GDM for pregnant women.

	OR (95% CI)	*p* value
Age	1.100 (1.020-1.180)	0.016
pre-pregnancy BMI	1.080 (1.002-1.170)	0.039
parity	0.410 (0.220-1.330)	0.006
FPG	4.260 (2.130- 8.490)	<0.001
HbA1c	4.480 (1.830- 11.010)	<0.001
the level of IgA	0.740 (0.500-1.100)	0.120
the percentage of B lymphocytes	1.080 (1.004-1.160)	0.037
the level of triglyceride	1.480 (1.010-2.160)	0.045
the level of progesterone	0.990 (0.980-1.001)	0.125
TPOAb	0.010 (0.370-2.740)	0.980

### Development and Validation of a Novel Predictive Nomogram

**Figure 1:**
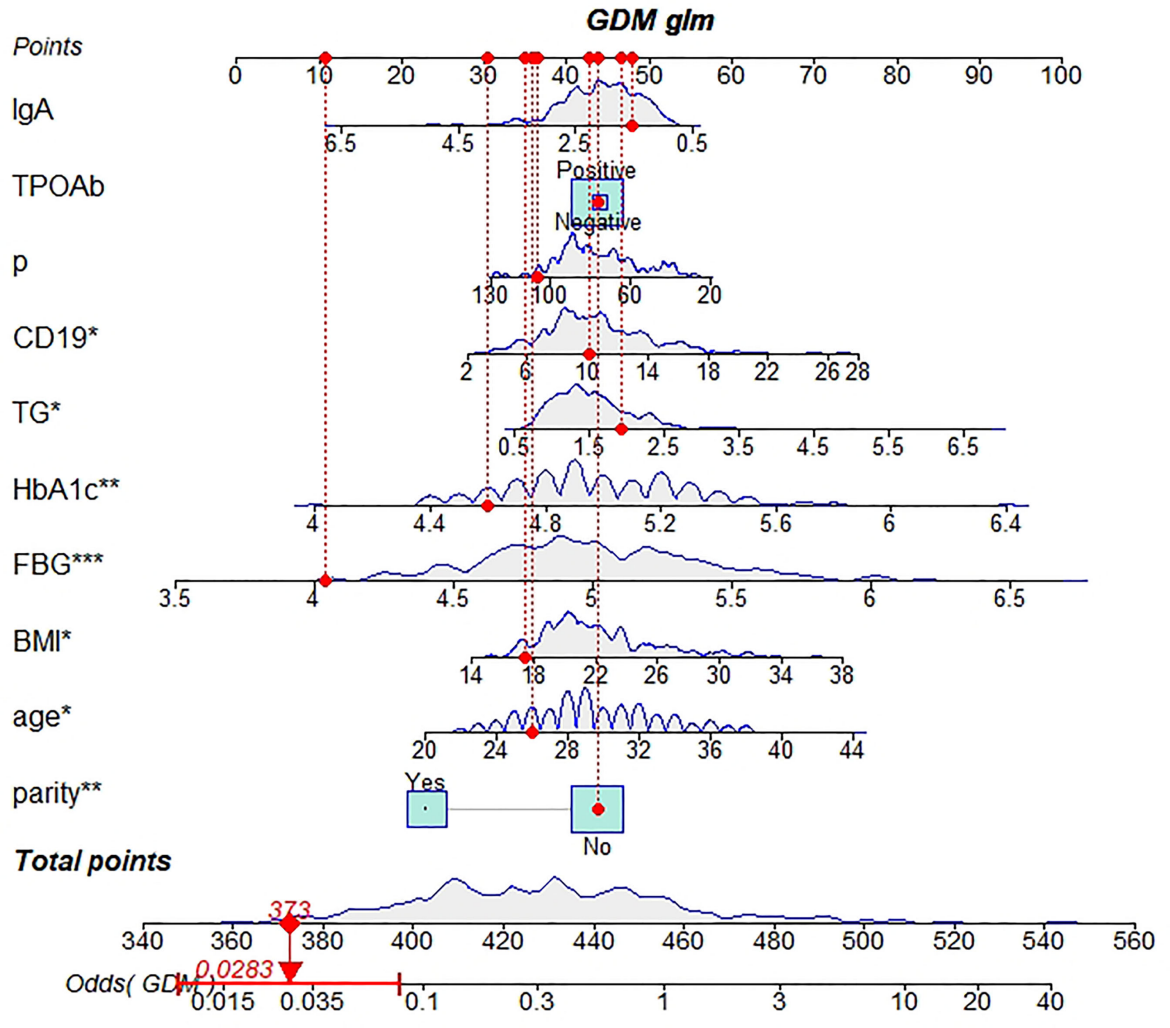
A novel nomogram predicting for the risk of GDM. Ten indicators in early pregnancy including age, pre-pregnancy BMI, parity, FPG, HbA1c, the level of IgA, the percentage of B lymphocytes, the level of triglyceride, the level of progesterone in early pregnancy and TPOAb were enrolled in the nomogram prediction model. The difference of the relative proportion of patients in each subgroup was represented by the area of rectangles. Patient 1 from our study is listed as an example (expressed in red). Her total score is 373 which indicating that her probability of GDM was 2.83%. GDM gestational diabetes mellitus, BMI body mass index, FPG fasting plasma glucose, CD19 the percentage of B lymphocytes, TG triglyceride. *P value between 0.01 and 0.05; **P value between 0.0009 and 0.009; ***P value < 0.0009.

**Figure 2:**
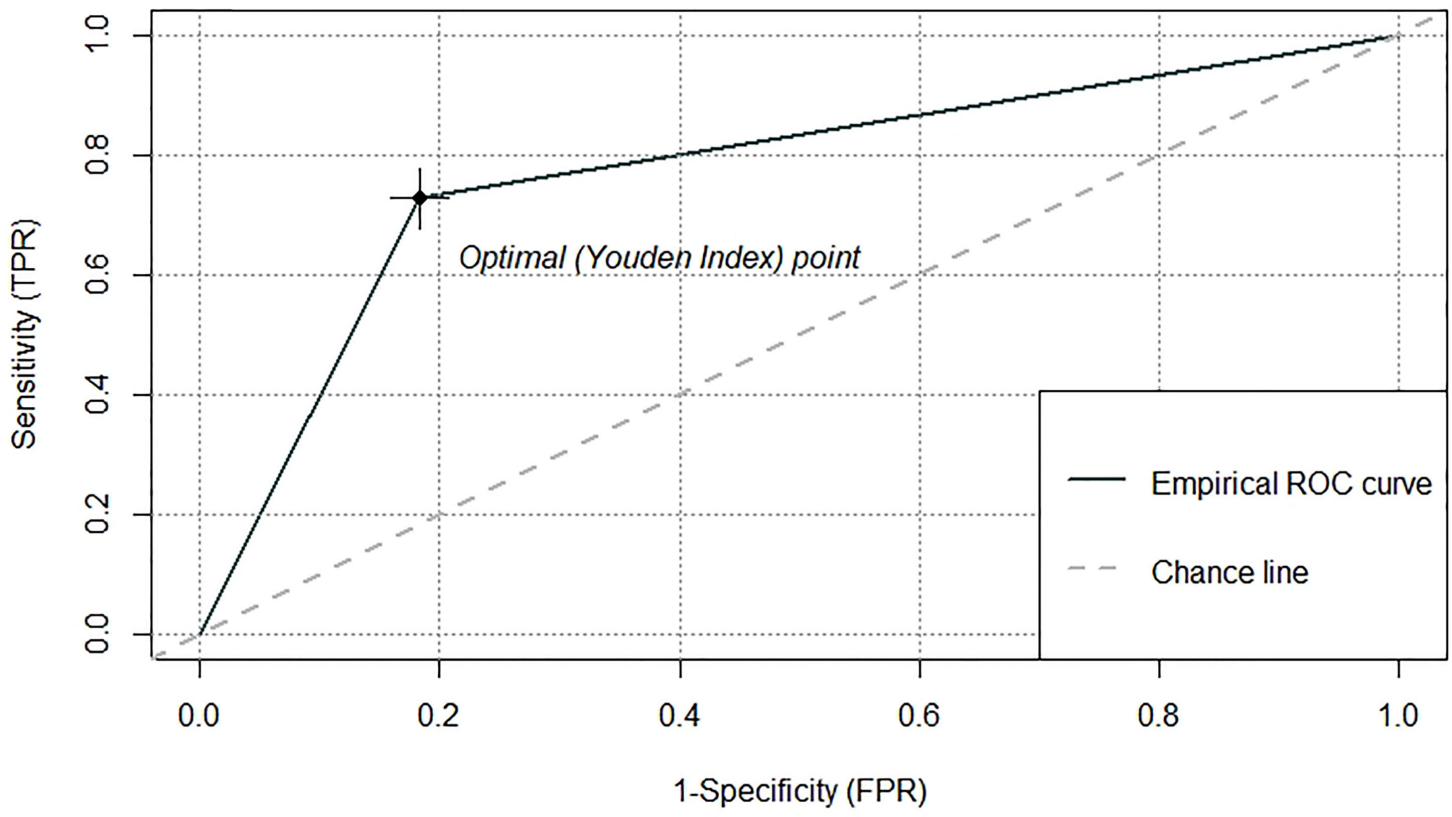
The ROC curve and the AUC value to evaluate the discrimination ability of the nomogram. ROC receiver operating characteristic, AUC area under the receiver operating characteristic curve.

**Figure 3:**
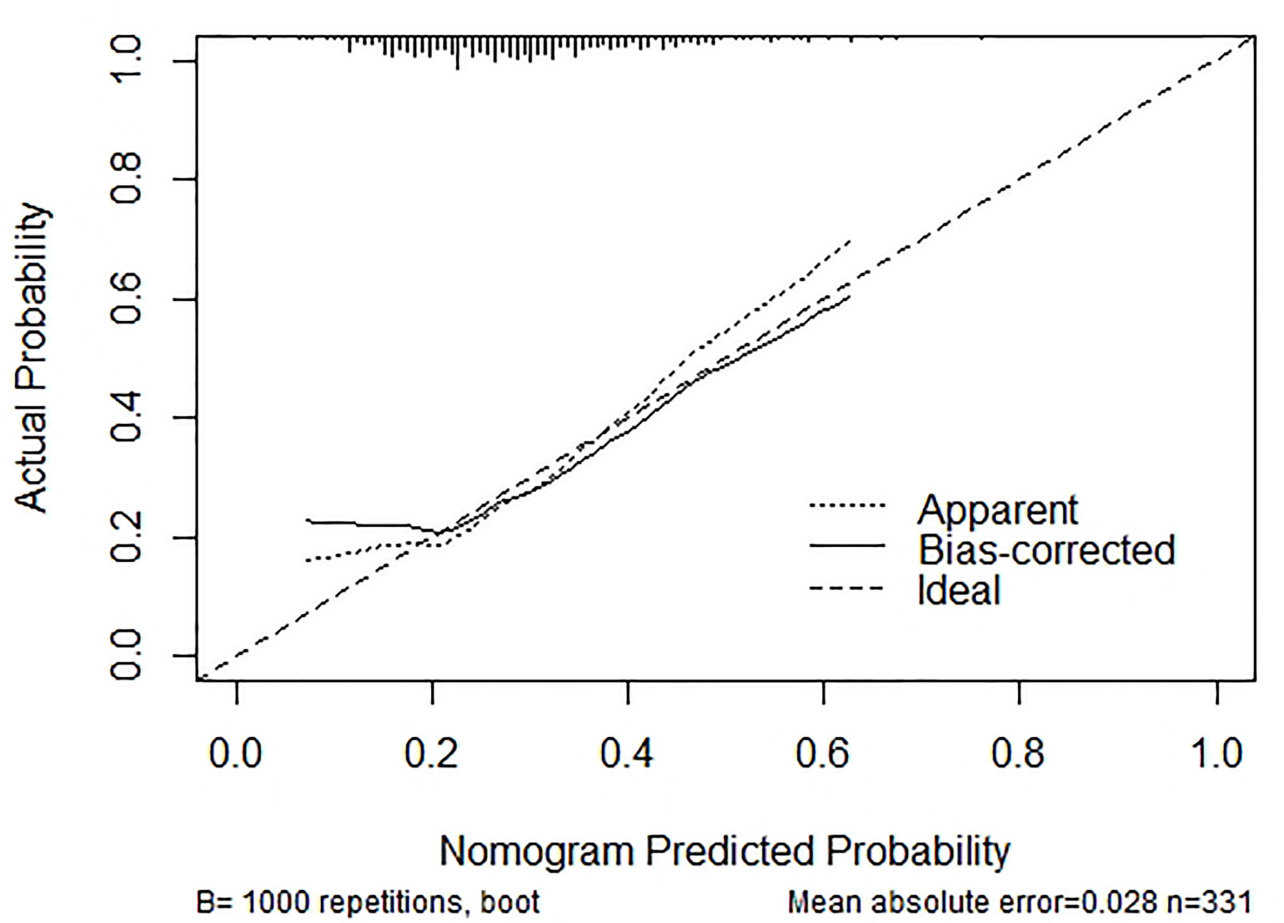
Calibration curves for the nomogram. The y-axis indicates the observed cumulative incidence for GDM, and the x-axis is the predicted probability of GDM based on the predictive model. The line adjacent to the ideal line represents the predictive accuracy.

**Figure 4:**
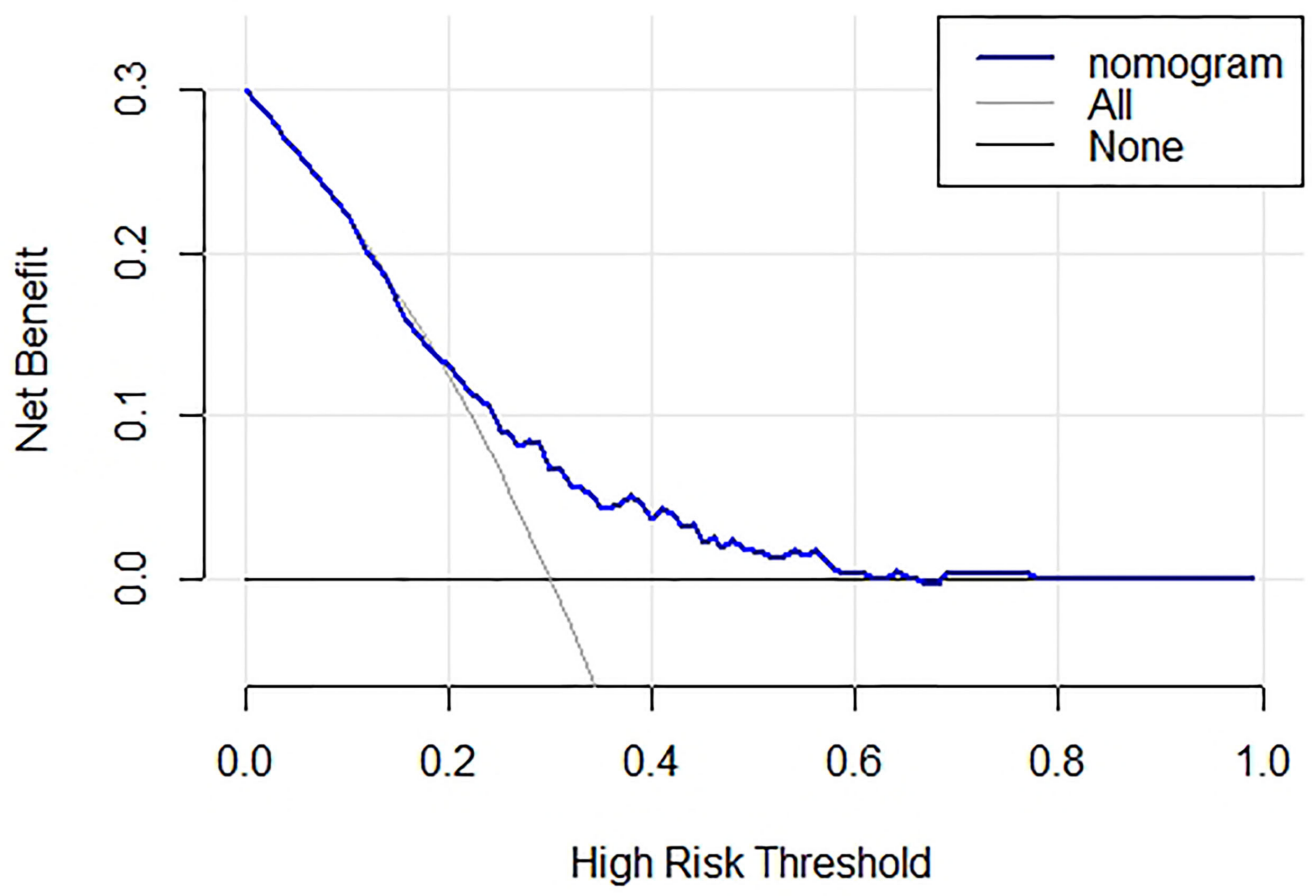
Decision curve analysis plot of the nomogram. Blue line represent the clinical net benefits according to the threshold probabilities; horizontal line assume no cases will experience GDM; grey line assume all cases will experience GDM. GDM, gestational diabetes mellitus.

Based on the outcomes of multivariable logistic model, the nomogram was constructed with 10 factors (**Figure 1**). We evaluated the discrimination of the nomogram by the ROC curve and AUC value. The AUC value was 0.772,95%CI 0.602-0.942 (*p*<0.001, **Figure 2**). The calibration curves demonstrate the agreement between the predicted values and the real outcomes (**Figure 3**). Hosmer-Lemeshow test *p* value was 0.198. At last, the DCA plot indicated good positive net benefits in the predictive nomogram model among majority threshold probabilities (**Figure 4**).

## Discussion

Early warning and early intervention are of great significance to the prevention, treatment and prognosis of GDM. In our study, a predictive nomogram for GDM in early pregnancy was established. The novelty of this study lies in (1) developing a nomogram integrating multiple factors such as demographic, immunity, lipid metabolism, hormone level and thyroid function to establish a GDM predictive model in early pregnancy, in particular, the introduction of the percentage of B lymphocytes and the level of IgA into the prediction model has not been reported before; (2) the nomogram demonstrated favorable discrimination and clinical usability for predicting the probability of GDM.

Our results are concordant with several previous findings reporting that the percentage of B lymphocytes in GDM patients were higher than the non GDM women (22, 23). Moreover, it was positively related to insulin resistance and increased in the first trimester of pregnancy in GDM patients. On account of B lymphocytes could act as antigen-presenting cells and influence immune reaction indirectly (24).Also, B lymphocytes promoted insulin resistance through modulation of T cells and production of pathogenic IgG antibodies (11). We speculate that the increase of the proportion of B lymphocytes may be an important driving factor in the pathogenesis of GDM. Similarly, in our previous study, the level of the percentage of B lymphocytes and IgA in the second trimester in GDM women was higher than normal ones (12). However, this study demonstrated that the IgA level in early pregnancy in women with GDM was lower. This change in IgA level suggests that B lymphocytes may have functional changes in different stages of pregnancy.

This study also integrated other classical indicators related to GDM to establish a predictive model in early pregnancy. Consistent with previous conclusions (25, 26), this analysis showed that the age, parity, pre-pregnancy BMI and baseline levels of triglycerides in GDM women were significantly higher than normal women. The level of triglyceride in the early stage was positively correlated with HOMA-IS in the second trimester of pregnancy. We also found the correlation between progesterone levels in early pregnancy and GDM, as Li M et al. reported (27). The early progesterone level of patients with GDM was significantly lower than the normal group and negatively correlated with insulin resistance in the second trimester of pregnancy. The post binding defect in insulin action during pregnancy is probably related to increasing amounts of progesterone (28). Therefore, progesterone level in early pregnancy plays an important role in GDM prediction.

In our study, there were not significantly difference in early pregnancy thyroid function between GDM group and normal women. Interestingly, thyroid antibodies TPOAb in early pregnancy was significantly correlated with fasting blood glucose in the second trimester of pregnancy, blood glucose 2 hours after taking sugar (*p* values are 0.007,0.009, respectively) and TGAb in early pregnancy was significantly correlated with glycosylated hemoglobin (*p*=0.003).The relationship between thyroid function and GDM is a controversial topic recently (29, 30). Some research revealed that Subclinical hypothyroidism (SCH) with positive antithyroid autoantibodies markedly increased GDM (3). The presence of thyroid autoantibodies can increase inflammatory factors and induce insulin resistance, which may also be one of the mechanisms by which it increases the risk of GDM (31, 32). While other study demonstrated thyroid diseases might not increase the risk of GDM in pregnant women (22). Given evidences indicates that thyroid antibodies are involved in the progress of GDM. We finally adopted TPOAb in the predictive model.

In contrast to existing models, our predictive nomogram is a valuable supplement which could improve the screening strategy for GDM. However, this study also has several limitations. First, the sample size and the number of GDM patients were limited. Second, data from single center might lack of representation for the whole pregnant population and the selection bias was inevitable. Third, the nomogram wasn’t validated in external database. Further multi-center prospective studies including dynamic monitoring in different gestational weeks should be carried out to update and validate the nomogram before widely clinical practice.

## Data Availability Statement

The original contributions presented in the study are included in the article. Further inquiries can be directed to the corresponding author.

## Ethics Statement

The studies involving human participants were reviewed and approved by the Shanghai General Hospital Institutional Review Board. The patients/participants provided their written informed consent to participate in this study.

## Author Contributions

YZ, MK, and XX designed this study. HZ, JZ, KH, J-YZ, JH, CL, JS, JW, and YY collected the data. MK conducted the statistical analyses. MK, YZ, and HZ drafted the manuscript. All authors contributed to this article and approved the submission and publishment.

## Conflict of Interest

The authors declare that the research was conducted in the absence of any commercial or financial relationships that could be construed as a potential conflict of interest.

## Publisher’s Note

All claims expressed in this article are solely those of the authors and do not necessarily represent those of their affiliated organizations, or those of the publisher, the editors and the reviewers. Any product that may be evaluated in this article, or claim that may be made by its manufacturer, is not guaranteed or endorsed by the publisher.
